# Editorials

**Published:** 1890-03

**Authors:** 


### HOMŒOPATHIC PHYSICIAN,

A MONTHLY JOURNAL OF

HOMŒOPATHIC MATERIA MEDICA AND CLINICAL MEDICINE.

If our school ever give up the strict inductive method of Hahnemann, we are lost, and deserve only to be mentioned as a caricature in the history of medicine."—CONSTANTINE HERING.

Vol. X. MARCH, 1890. No. 3.

### EDITORIALS.

ARGUMENT AND REASONING.—" We must meet them as gentlemen, and by argument and reasoning show on what false ground they stand. We must convince them by sound reasoning that medicine is a much broader field than Homœopathy, for it covers *every* pathy; and, avoiding the vituperative style of many of Hahnemann's followers, we should prove the narrow-mindedness of his system."

Thus an allopath (he will not own that name, however) calmly considers the position of the followers of Hahnemann. We have repeatedly seen the results of attempts that have been made to convince " by argument and reasoning " against the law of the similars. Dr. Hering first became acquainted with Homœopathy in an endeavor to write it down by " reasoning." He wrestled with the angel and the angel did not come out of the conflict second best. Every Hahnemannian can appreciate the result of that wrestling match in the works of Constantine Hering.

Dr. P. P. Wells, of Brooklyn, our honored venerable colleague (may he continue with us many years more), had a similar experience, only to find that the reasoning was all on the side of Homœopathy.

If it would avail, we should like to ask, from whom has vituperation and abuse come? Homœopathy has been mostly on the defensive since Hahnemann first promulgated his discoveries. If the writer quoted above would only read Ameke's *History of Homœopathy*, he would find that *odium medicum*

has been, and continues to be the stock in trade of allopaths, and of them alone. It is true that Hahnemannians have always decried mongrelism, and it is to be hoped they will continue doing so, for to do this means only to traduce dishonesty.

Reasoning will not profit allopathy. Homœopathy can go beyond reasoning and produce results which no other school of medicine can approach. These results have made Homœopathy what it is to-day—a blessing to mankind.

---

The same writer says: "Homœopathy is not broad enough, but its followers cling to their tattered standards, believing that they must enlist under the banner of some school and follow some pathy, whereas their energies could be much more profitably employed in healing the sick by the use of any and all agencies known than in trying to conform to a single theory, or trying to make the people believe that they do."

If the gentleman will make an honest attempt to put to practical test this Homœopathy—which is based on an immutable law of Nature—he will find it sufficiently broad to cure any curable case of any disease. But the trial must be an honest one.

"Its followers cling to their tattered standards." Yes, they do "cling," and the "tattered" condition of their "standards" shows what battles they have fought and won. Whenever allopathy has met Homœopathy in the open field, which has been the victor? Homœopathy has always been, and is ever ready to put to actual test the merits of the two schools; but allopathy will never meet the trial.

"Believing that they must enlist under the banner of some school and follow some pathy." They do believe "that they must enlist under the banner of some school." This belief, however, is firmly founded not upon mere conviction, but upon thought, and upon the "reasoning" which the gentleman claims as *his* school's sole possession. The "banner" under which the Hahnemannian enlists is inscribed with this motto: "HOMŒOPATHY, SCIENTIFIC MEDICINE, EXCELSIOR." And the path which he follows is the path of glory, made glorious by the lives he saves, and not the path which "leads but to the grave"—the allopath.

"Their energies could be much more profitably employed in healing the sick by the use of any and all agencies known." Our dear friend in that clause shows plainly his ignorance of Homœopathy. Were he possessed of but a superficial knowledge of Hahnemann's teachings he would know that our every energy is directed toward "healing the sick by the use of any and all agencies known." But before the Hahnemannian attempts to use "any and all agencies" he brings the only law of therapeutics to his aid, and he can then use these "agencies" in in a rational, sane manner. He has the light of this law to guide him, and he does not flounder in the darkness of the Middle Ages.

"Trying to conform to a single theory, or trying to make the people believe that they do."

Wrong again, dear friend. Must we again repeat that we have a LAW? This will bear repetition, if but for the simple reason that an allopath knows or seems to know nothing of it. People, generally, give but little thought to theory in medicine. They wish to be cured of their ailments, and millions have learned that when they wished to be cured speedily, safely, and permanently they need only call on a follower of Hahnemann—and they do not call in vain!

Again, in the same article, we read: "No theory yet discovered explains *how* drugs cure diseases, neither do we know *how* soul and body are united, and it is not necessary that we should know. But all that we can know certainly, all that assiduous observation can teach us is, that the cure of such a disease succeeds more or less constantly the administration of such a remedy."

Has any Hahnemannian ever claimed to know "*how* drugs cure diseases"? We are aware that there are hangers-on to the bright robes of Homœopathy who profess to have omniscience, but these men are greater (in their own estimation) than even Hahnemann himself. If we can read language in which there seems no ambiguity, certainly those who make this claim are of the allopathic school. Do we not hear of them prating about this drug paralyzing the respiratory centres, and of that being inhibitory of vaso-motor nerves, etc., etc.? And, having such powers, do they not prattle about what they can do with these agents? And yet they set themselves up as being able to define a Hahnemannian's position!

---

And "all that we [allopaths] can know certainly, all that assiduous observation can teach us is [note this, Hahnemanians], that the cure of such a disease succeeds more or less constantly the administration of such a remedy." Fancy Homœopathy having nothing better than this to offer: "that the cure of such a disease succeeds more or less constantly the administration of such a remedy." And they call this scientific medicine!

What is defined as scientific? "Agreeing with or depending on the rules or principles of science." What is science? "Science consists in an infallible and unchanging knowledge of phenomena." Empiricism is an unreasoning and instinctive imitation of previous practice." Will a second glance be required to show one who knows even the least of homœopathic therapeutics, and even more of allopathic therapeutics, which is the scientific and which the empirical? G. H. C.

---

THE DIFFERENCE BETWEEN THE OLD SCHOOL AND THE NEW.—A follower of Hahnemann needs no better argument for his attitude against eclecticism and allopathy than is found in any old-school journal. If one had had experience in putting to actual test the law of the similars, he is not in need of hyper-acute vision in order to see that scientific (?) medicine is usually floundering in the dark in respect of the treatment of disease.

We cannot dwell too forcibly on the difference between old-school empiricism and homœopathic law. Illustration of this difference is clearly manifest in the old-school's present method of treating various forms of spinal affections, particularly sclerosis. It will be remembered that a few years ago a Russian surgeon, while suspending an ataxic patient for the purpose of adjusting a plaster jacket, found that the ataxic symptoms were less prominent after the suspension. This he published for his medical brethren. Instantly from one end of the medical (old-school) world to the other went up the cry, "The treatment for locomotory ataxia is suspension." What a scientific foundation for a fact in scientific medicine! Then their journals were filled with the good effects of suspension.

But, like all their other good things, this seemed to be only a fad, and now we rarely see anything on the subject. However, a recent issue of the *Lancet* contains a series of twenty-one cases treated by suspension. A summary of results shows that not one case received any benefit, and that some were made worse. Is there any follower of Hahnemann who, after treating twenty-one cases of any affection, by adhering to the only law of therapeutics and failed to see any good results, is there one who would have the audacity to believe there was any good in that law? We cannot believe that one such person exists. If results had not shown the truth of the law there would be no Homœopathy, and the world would then be at the mercy of allopathy, as it was in the Dark Ages, and the bright beacon of Homœopathy would be used only by wreckers, many of whom are now using its brilliant light to blind the public to mongrelism under the name of progressive Homœopathy.

### The Unattainable.

### A Song of the Unattainable.

"For the few-and-far-between,
For the very-seldom-seen,
For the un-catch-hold-uponable I sigh!
The unclutchable I'd clutch,
The untouchable I'd touch,
For the ungrabbed and ungrabable I die!

"Oh! I burn and sigh and gasp
For the just-beyond-the-grasp,
For the far-unovertakable I yearn;
And the vulgar here-and-now
I ignore and disavow,
And the good-enough-for-others how I spurn!

"Oh! how I moan and screech
For the just-beyond-the-reach,
The too-far-away-to-grab I would ensnare,
The ungainable I'd gain,
The unattainable attain,
And chase the un-catch-onto to its lair."

The unattainable is always being sought by those who feel themselves above ordinary mortals. And even though they claim to "catch on" to it, lookers-on dare not deny it, if they value peace, and do not wish to be deluged with mere assertions which are offered as arguments. When this desire takes the form of attempting to show that the product of a disease is a curative of that disease, and that it is only necessary to diagnose the disease as being present, and then administer one dose, or more, of its product in order to cure it, then it is time to say that is mere assertion, and we wish the proof.

The onus of proof is most upon him who affirms. It were "a consummation devoutly to be wished" if the treatment of disease could be so simplified, and we do not deny that it may be true; but we have never seen even one case that would go to show it to be true. In our present light, before we may use the various morbid products intelligently, we must proceed as we do with other substances, prove them. Then, and only then, can we feel we are on safe ground. The true follower of Hahnemann will not theorize in respect of the power of any so-called curative. He has an infallible way of learning just what each is capable of doing, and he must follow in that way rigidly. True, by induction he may assume that a certain drug, or other substance, ponderable or imponderable, may possess certain powers; but he is not able to know until the proper course has been pursued that his claim is well founded.

The literature of our school abounds with examples of this kind, and he who has put them to the test has found that he is a victim of misplaced confidence, and has been glad to hasten back to law.

The same applies to mixed remedies. As homœopathicians, claiming to and knowing that we do possess an unerring guide which leads us to know what remedies can do, we certainly need more than prodding when we run after *ignis fatui*, or, as Dr. Hering put it, "We should not leave an old friend in order to follow a coquette."

A remedy is a remedy, even though it be composed of one hundred or five hundred different ingredients, provided it has been proved homœopathically. Until this is done, even though we use the simplest element, we can know nothing of its powers, and if we attempt to use it without a proving we are guilty of lawless empiricism. G. H. C.

---

The Minuteness of the Dose.—"I do not consider any as my followers, who, in addition to leading an irreproachable, perfectly moral life, do not practice the new art in such a manner that the remedy he administers to the patient in a non-medicinal vehicle (sugar of milk or diluted alcohol) contains such a small subtle dose of the medicine that neither the senses or chemical analysis can detect the smallest absolutely hurtful medicinal substance; indeed, not the slightest trace of anything medicinal at all, which presupposes a minuteness of dose that must indubitably dispel all anxiety from all officers of state who have to do with medical police."—Hahnemann, 1820.

---

Quinine.—A journal in Boston estimates that the people of that place consumed about a ton of quinine in the course of ten days while la grippe was at its height. Just about four grains a day for each man, woman, and child. About this time look out for an epidemic of quinine insanity. Yet quinine will not be credited with this dethronement of reason. On the contrary, it will be considered a freak of la grippe. G. H. C.

---

La Grippe.—In response to our request we have received a number of papers treating on the late pandemic, "la grippe," a few of which we have published. As we expected, homœopathic treatment has been successful, and no sequelæ have been noticed. In contrast to this, old-school journals are now publishing the results of their treatment, and the published mortality lists show what allopathy has done—for the undertakers.

Some of our correspondents speak of old troubles, which had been suppressed by drugs, reappearing after homœopathic treatment. This goes to prove Hahnemann's observations correct. In all cases we can unhesitatingly promise these patients that if they continue the treatment for awhile all their old symptoms will disappear, and they will be in a better state of health than since the original attack was suppressed.

---

INTERMITTENT FEVER TREATED BY FRICTION.—Some more scientific (?) therapeutics from the *Lancet*: "Alois Fênykövy communicates to a Vienna medical journal an account of some observations made on the treatment of intermittent fever by means of friction of the back along the spine. Many years ago, while at Nisch with his regiment, there occurred so many cases of intermittent fever that the stock of quinine was becoming exhausted, and, in order that the patients might not be entirely without some sort of treatment, it was ordered that they should be rubbed twice a day along the spine with simple ointment. The day after this order had been given it appeared that the usual attack had not come on; *accordingly* (our italics), since that time Dr. Fênykövy has very frequently employed this treatment, and usually with marked success. Indeed, he says that three-fourths of his cases have done very well without any quinine at all.

We pass this on for the benefit of those "progressive" (mongrel) homœopaths who are always clamoring about the necessity of quinine in intermittents. We trust the source will be sufficiently regular and scientific to satisfy even the most blatant.

G. H. C.

---

THE DEFINITION OF A HOMŒOPATHIC PHYSICIAN.—In the January number, at page 5, we called attention to the surprising definition of a homœopathic physician, given by one of the most prominent homœopathic physicians in New York City.

Our friend and subscriber, Dr. A. McNeil, of San Francisco, whose interesting contributions to our pages are familiar to all our readers, in a recent letter to us commenting upon the New York definition, gives this clever illustration of its absurdity:

"The definition of a Christian is one who is a member of 'our Church.' No matter if I have recourse to a violation of all the commandments, I am a Christian so long as I am a member of our Church. As all the members are like me, they will not turn me out." W. M. J.

